# Supplementary material for: Demonstration of angular-momentum-resolved electron energy-loss spectroscopy
Source: Nat Commun. 2025 Jul 17;16:6601. doi: 10.1038/s41467-025-60804-3 (PMC12271546; doi:10.1038/s41467-025-60804-3)
Supplement: Supplementary file 1 — Supplementary Information [file 41467_2025_60804_MOESM1_ESM.pdf]

Supplementary Information

Demonstration of angular-momentum-resolved electron energy-loss spectroscopy

A. H. Tavabi,[1†] P. Rosi,[2†] G. Bertoni,[2*] E. Rotunno,[2*] L. Belsito,[3] A. Roncaglia,[3] S. Frabboni,[2,4] G. C. Gazzadi,[2] E. Karimi,[5] P. Tiemeijer,[6] R. E. Dunin-Borkowski,[1] V. Grillo[2]

[1] Ernst Ruska-Centre for Microscopy and Spectroscopy with Electrons, Forschungszentrum Jülich, 52425 Jülich, Germany

[2] Istituto Nanoscienze, Consiglio Nazionale delle Ricerche, Via G. Campi 213/A, 41125 Modena, Italy

[3] Istituto per lo Studio dei Materiali Nanostrutturati, Consiglio Nazionale delle Ricerche, Via P. Gobetti 101, 40129 Bologna, Italy

[4] Università di Modena e Reggio Emilia, Via G. Campi 213/A, 41125 Modena, Italy

[5] Department of Physics, University of Ottawa, Ottawa, Ontario K1N 6N5, Canada

[6] Thermo Fisher Scientific, PO Box 80066, 5600 KA Eindhoven, the Netherlands

† Equally contributing authors

* Corresponding authors. e-mail: giovanni.bertoni@cnr.it, enzo.rotunno@cnr.it

Table of Contents

Supplementary Note 1. Further experimental details. S3

Supplementary Figure 1. STEM results from h-BN edge region. S3

Supplementary Figure 2. Results from NMF decomposition. S4

Supplementary Note 2. Mixed dynamic form factor approximation. S4

Supplementary Note 3. Further details of the Monte Carlo simulations. S5

Supplementary Figure 3. Representative description of a multislice simulation of inelastic scattering. S7

Supplementary Figure 4. Comparison between 1s Bloch state approximation and full multislice calculation of inelastic scattering. S8

Supplementari Note 4. Details of the chromatic effects of the sorter. S8

Supplementary Figure 5. Effects of chromatic aberration. S9

Supplementary Note 5. Quantitative evaluation of the quality of OAM decomposition. S10

Supplementary Note 6. OAM-EELS experiment in ideal experimental conditions. S11

Supplementary Figure 6. Simulated OAM profiles in the case of negligible drift. S11

Supplementary References S12

Supplementary Note 1. Further experimental details.

In order to further confirm the ability to map OAM features, Supplementary Figure 1 shows results obtained from an edge region of h-BN in scanning mode. This 15×11 pixels EELS dataset was pre-processed as the data in the manuscript, then followed by k-means clustering algorithm by selecting subsets of points in the OAM domain. Two regions were identified, corresponding to the bulk and the edge of the sample, with slightly different OAM profiles. Increased OAM broadening in the edge region may result from the presence of defects or from folding (i.e., misorientation) of the sample edge. In this experiment, we wanted to verify if a decomposition in OAM components can be obtained without *a priori* knowledge of the physics of the process by using blind methods, as in statistical multivariate analysis[1]. Such methods have the advantage that neither the psf from the zero-loss spectrum nor simulated OAM profiles after scattering are required, at the expense of uncertainty in the physical interpretability of the results. We used non-negative matrix factorization (NMF) to provide positively-defined OAM components (Supplementary Figure 2). Before applying NMF, the dataset was symmetrized in OAM around $\ell = 0$ to increase signal to noise ratio, permitting to extract the principal components from their variance with respect to the baseline from noise or small artifacts, as presented in the hierarchical plot of variance (scree plot) of Supplementary Figure 2a. Two principal components (NMF 1 and NMF 2) are discerned, and their EEL spectra and OAM profiles are shown in Supplementary Figures 2b and 2c, respectively. NMF 1 is centred at $\ell = 0$ and has enhanced features from the expected π* transition to the unoccupied density of states (as the first peak at the onset). However, there are also contributions at higher energies due to residual superposition with the σ* transitions. NMF 2 has clear fingerprints of the transition to sp_2 empty states (σ*). However, the large ℓ variance due to the low probe convergence semi-angle in the experiment (~1.8 mrad) does not permit clear separation of the two components at small ℓ.

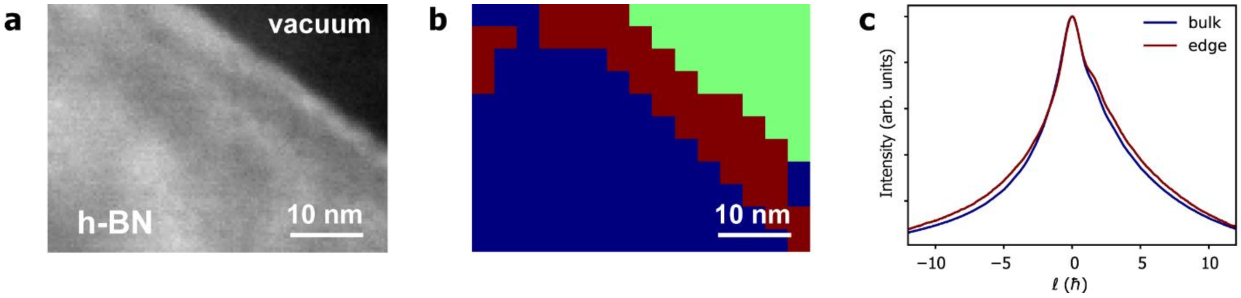

Supplementary Figure 1. STEM results from h-BN edge region. a Annular dark-field image recorded from the investigated h-BN edge region. **b** k-means clustering of the OAM-EELS dataset from the area shown in **a** (bulk is blue, edge is brown, and vacuum is green). **c** OAM profiles from the bulk (blue line) and edge (brown line) sub-regions in **b**.

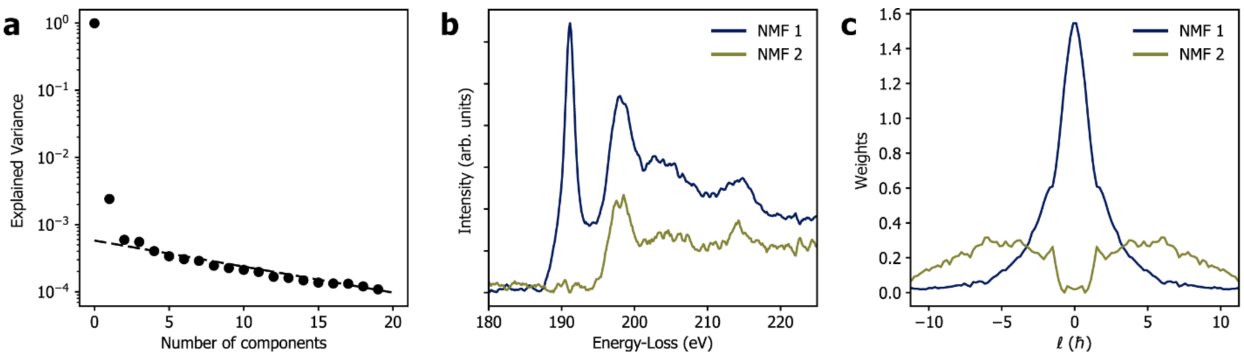

Supplementary Figure 2. Results from NMF decomposition. a Scree plot of the variance after decomposition of the spectrum from the blue region shown in Supplementary Figure 1. **b** Extracted EEL spectra of the first two components NMF 1 (dark blue line) and NMF 2 (olive green line). **c** Corresponding OAM profiles of components NMF 1 (dark blue line) and NMF 2 (olive green line).

Supplementary Note 2. Mixed dynamic form factor approximation.

On the assumption that the electron is initially in a pure state described by the density matrix $\rho_0(k_i, k_i) = |\psi_p >< \psi_p|)$ and that a single scattering event occurs on a single atom, the final electron subsystem can be described by a reduced density matrix ρ in the form[2]:

$$\rho(k_f, k_f') = \frac{S(\mathbf{Q},\mathbf{Q}',\omega)}{Q^2 Q'^2} * \rho_0(k_i, k_i'), \quad (1)$$

where $\mathbf{Q} = (\mathbf{k}_f - \mathbf{k}_i, q_E)$ and $\mathbf{Q}' = (\mathbf{k}_f' - \mathbf{k}_i', q_E')$ are 3D vectors and account for kinematical effects of the exchange of momentum $\mathbf{k}_f, \mathbf{k}_i$ and energy through $q_E = E/(\hbar v)$, E is the energy loss, v is the electron velocity, $\hbar$ is the reduced Planck constant and q_E is related to the characteristic scattering angle by the relation $q_E = 2\pi\theta_E/\lambda$, where λ is the electron wavelength, such that $\theta_E \cong \frac{E}{2E_0}$, where E_0 is the electron beam energy. The symbol $*$ indicates convolution in reciprocal space and all the atomic details are included in the mixed dynamic form factor $S(\mathbf{Q}, \mathbf{Q}', \omega)$.

The $1/Q^2$ dependence adds a Lorentzian-like envelope to the dependence on exchanged momentum $\mathbf{q} = \mathbf{k}_f - \mathbf{k}_i$, which concentrates the scattering angle within θ_E. In the dipole approximation for highly symmetrical systems, $S(\mathbf{Q}, \mathbf{Q}', \omega) \approx a\mathbf{Q} \cdot \mathbf{Q}' + b$ can be written in the form[3]:

$$\frac{S(\mathbf{Q},\mathbf{Q}',\omega)}{Q^2 Q'^2} = \frac{a\mathbf{q}\cdot\mathbf{q}' + c}{(q^2+q_E^2)(q'^2+q_E'^2)} = \frac{aqq'\cos(\Delta\phi)+c}{(q^2+q_E^2)(q'^2+q_E'^2)} = \frac{a\,qq'\exp(i\phi)\exp(-i\phi')}{2(q^2+q_E^2)(q'^2+q_E'^2)} + \frac{a\,qq'\exp(-i\phi)\exp(i\phi')}{2(q^2+q_E^2)(q'^2+q_E'^2)} + \frac{c}{(q^2+q_E^2)(q'^2+q_E'^2)}, \quad (2)$$

where a and b depend on energy and $c = aq_E q_E' + b$. The Fourier transform provides the mixed dynamic form factor in direct space. For this calculation, it is convenient to Fourier transform each term separately.

In many cases, we measure only OAM and the relative coherence of these terms is less important. We then Fourier transform the two factorized functions in the q, q' variables. The problem becomes one of finding the inverse Fourier transform of the function:

$$\mathrm{g}(\mathbf{q}) = \frac{aq^n \exp(in\phi)}{q^2+q_E^2}. \tag{3}$$

The inverse Fourier transform with respect to separate terms is based on a 0th and 1st order Hankel transform through the formulae:

$$\mathrm{H}_0\left\{\frac{c}{q^2+q_E^2}\right\} = c\mathrm{K}_0(q_E r), \tag{4}$$

$$\mathrm{H}_1\left\{\frac{aq}{q^2+q_E^2}\right\} = aq_E\mathrm{K}_1(q_E r). \tag{5}$$

These result in the atomic functions:

$$\mathrm{f}_{a,0}(\mathbf{r}) \approx c\mathrm{K}_0(q_E r), \tag{6}$$

$$\mathrm{f}_{a,\pm 1}(\mathbf{r}) \approx aq_E \exp(\pm i\phi_a)\mathrm{K}_1(q_E r). \tag{7}$$

It should be noted that the function $\mathrm{K}_n(q_E r)$ is divergent at $r = 0$, due to the limits of the approximations for S, especially when not considering the radial degrees of freedom. A common but still approximate way to account for these limits is to impose a cutoff at $r_c = \frac{\lambda}{\sqrt{2\theta_E}}$. For the case $m = -1, +1$, we added a linear increase in amplitude of the form $\mathrm{f}_{a,m}(r) \approx \frac{r}{r_c}$ for $r < r_c$.

The use of an incoherent Monte Carlo calculation is justified by the assumption of independent atomic scattering. In this approximation, the density matrix of the system is an incoherent average of the density matrix of scattering at each atom, in the form:

$$\rho_{\mathrm{tot}}(k_f, k_f') = \frac{1}{N}\sum_{i=1}^{N} \rho_i(k_f, k_f'). \tag{8}$$

Supplementary Note 3. Further details of the Monte Carlo simulations.

Here, we address the numerical simulation of inelastically scattered electrons in a crystalline lattice, focusing on the computation of the wavefunction at the exit plane of the sample, which is a necessary step for accurate interpretation of the orbital angular momentum spectra presented in the main text. An accurate treatment of inelastic scattering in the TEM introduces significant complexities. Unlike elastic scattering, inelastic scattering results in a loss of coherence, requiring an incoherent summation over all illuminated atomic positions to accurately capture the wavefunction dynamics.

Such an incoherent summation has been examined rigorously in previous works, notably using multislice method[4]. These approaches involve dividing the sample into a series of finely resolved

layers, with each layer contributing a unique scattering potential that sequentially modulates the electron wavefront (multislice simulation). In a multislice framework, inelastic scattering is computed as a product of the incident wavefunction propagated to the depth of the scattering atoms and the atomic inelastic scattering factors, which are described by the two functions $\mathrm{f}_{a,0}(\mathbf{r})$ and $\mathrm{f}_{a,\pm1}(\mathbf{r})$. Each scattered wavefunction from the individual atoms is then propagated to the exit surface of the sample using a multislice simulation. The final wavefunction at the exit surface (called exit wave) is obtained as an incoherent sum (as a sum of the squared moduli of the scattered waves from each atom), providing an accurate model of the total inelastically scattered wavefunction.

In the present work, several experimental constraints preclude the direct application of a full multislice approach. The long acquisition times required for the experiments lead to unavoidable sample drift, resulting in an uncertain and variable probe position relative to the sample, which prevents precise localization of the probe. To accommodate this uncertainty, we implemented a Monte Carlo simulation that samples continuously across the atomic coordinates over the illuminated region, providing a robust statistical model for probe drift and position averaging.

The analysis is further complicated by the small convergence angle of the electron beam, together with a slight defocus, which expands the probe size beyond that used in conventional atomically resolved STEM. The enlarged probe, coupled with the incoherence intrinsic to inelastic scattering, renders a full multislice simulation computationally prohibitive.

The simulation shown in Supplementary Figure 3 serves as an illustration of inelastic scattering from a single atom in a crystalline sample modelled through a multislice approach. For simplicity, we consider inelastic scattering from a B atom in the middle of the sample along z. In this setup, a defocused, large-convergence-angle electron probe, which is not specifically aligned with any atomic column, propagates through the sample until it reaches the mid-depth point. At this point, the inelastic scattering event is triggered, and the simulation continues by tracking only the inelastically scattered wavefunction to the sample exit plane. The simulation compares two scenarios for the inelastic scattering process, one with an orbital angular momentum transition of $m = 0$ (left) and the other with $m = +1$ (right).

As a result of the thin nature of the sample (< 10 nm), a kinematic approximation can be used to describe the exit wave, known as the 1s Bloch state approximation. In this approximation, the interaction of the electron probe with the periodic crystal potential is approximated by considering only the dominant Bloch state of the crystal. Specifically, the 1s Bloch state represents a strong channelling mode that localizes the electron density along the atomic columns. For a sufficiently thin sample, this state effectively describes how the electron beam interacts with the crystal potential, capturing the main contributions of the channelling effect without requiring a full multislice treatment for each atomic layer.

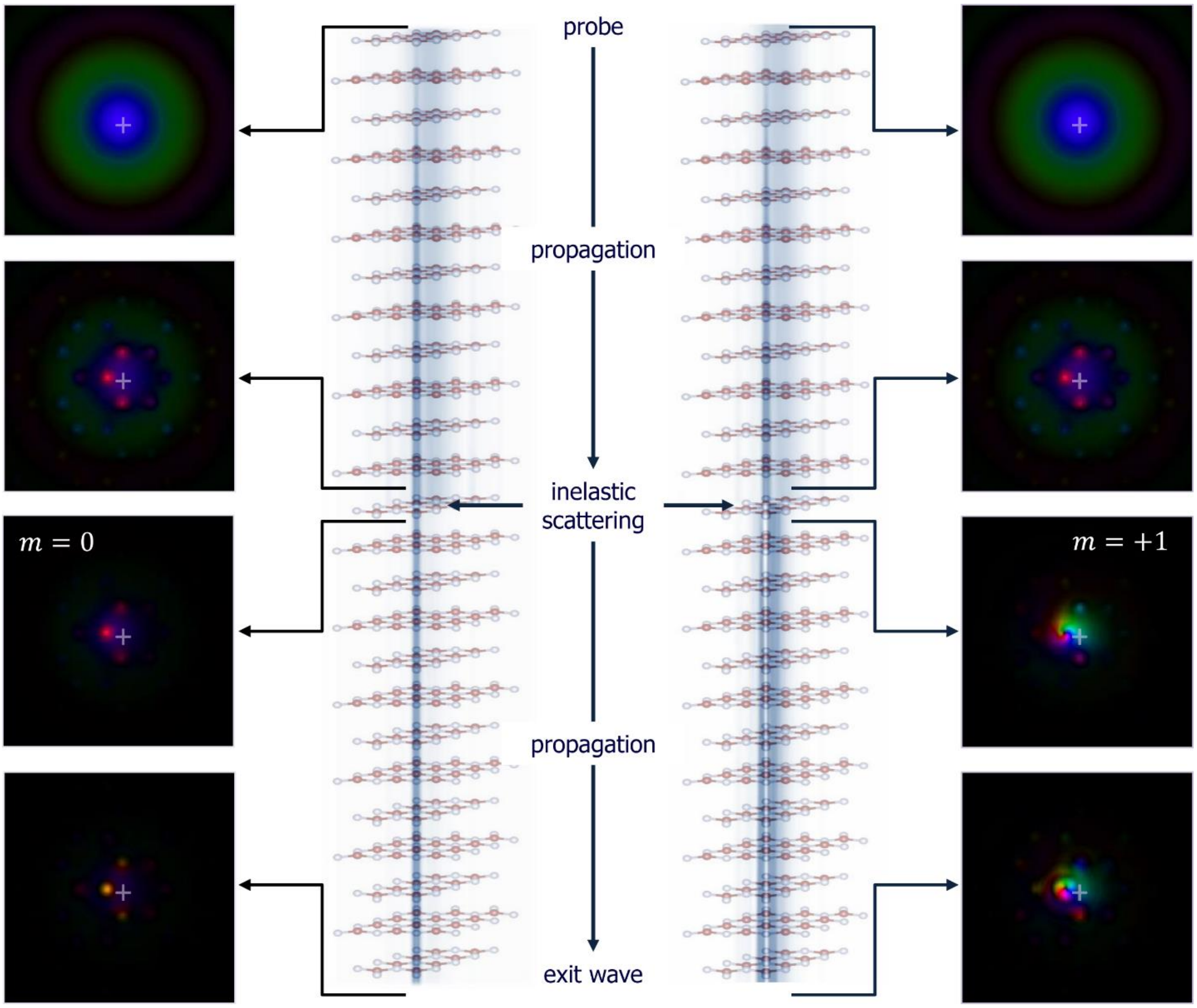

Supplementary Figure 3. Representative description of a multislice simulation of inelastic scattering. Example of scattering from a single atom located at the centre of a crystalline sample. The left and right columns show simulations for transitions with orbital angular momentum values $m = 0$ (left) and $m = +1$ (right), respectively. A large, defocused probe, not centred on a specific atomic column, is propagated halfway through the sample before an inelastic scattering event occurs at the atom position. (The cross indicates the probe centre at the entrance of the sample). From this point onward, only the scattered wave is followed, resulting in a marked contrast change. The abrupt shift in contrast highlights the transition from elastic to inelastic propagation. (The scattering atom is near to the cross on the left side).

Under this approximation, the exit wavefunction can be simplified as a product of three main components: the incident probe wavefunction, the scattering cross-section of the atom (representing the probability of an inelastic transition at the atom position) and the 1s Bloch state of the crystal, as show in Supplementary Figure 4. A further Gaussian envelope ($\sigma = 0.32$ nm) centred

on the scattering atom has been found to qualitatively improve agreement with the multislice simulation.

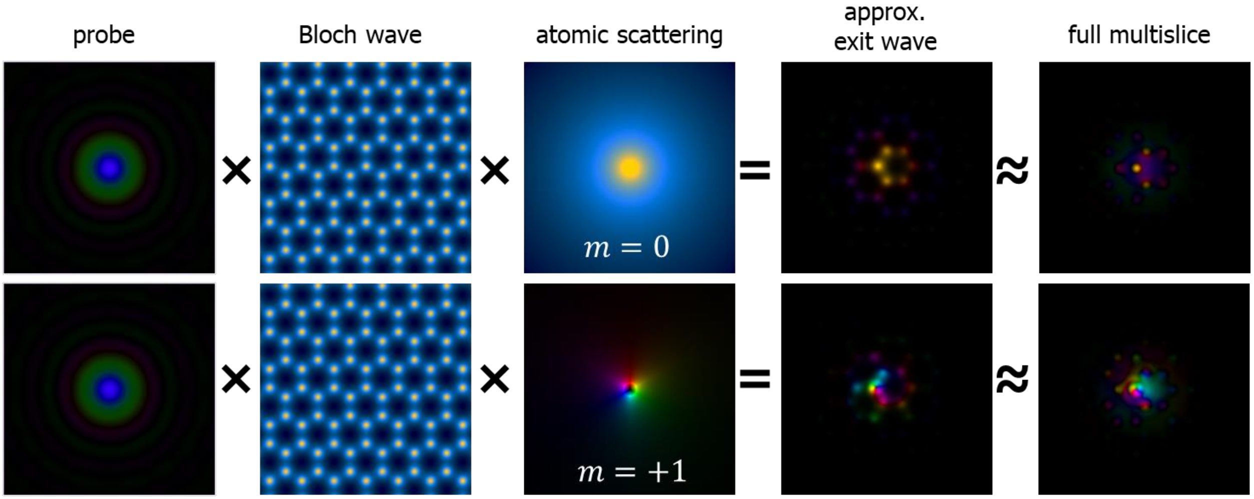

Supplementary Figure 4. Comparison between 1s Bloch state approximation and full multislice calculation of inelastic scattering. The upper row shows a transition with orbital angular momentum $m = 0$, while the lower row shows a transition with $m = +1$. In the simplified model (left to right), the incident probe is multiplied by the 1s Bloch wave of the crystal, which accounts for the channelling effect and the atomic cross-section for inelastic scattering.

This approach not only simplifies the numerical treatment of the inelastic scattering process but also yields an exit wave that accurately reflects the channelling dynamics of the probe through the crystal, which is governed primarily by the 1s Bloch state. The remarkable resemblance of the simplified model to the computationally intensive multislice calculation demonstrates the effectiveness of the 1s Bloch state approximation in accurately capturing the essential physics of elastic scattering and propagation.

By using this approximation, it is computationally feasible to run a Monte Carlo simulation for different probe positions and scattering atomic positions. The Hankel transforms of these approximated exit waves provide the OAM spectra, which are shown in Figure 3 of the main text and used for fitting in Figure 4 of the main text.

Supplementari Note 4. Details of the chromatic effects of the sorter.

Supplementary Figure 5 describes the influence of chromatic aberration on the OAM Sorter's 2D psf (top row) and 1D psf (bottom row). We recall that the energy spectrometer's optics integrate the beam along the direction orthogonal to the OAM (i.e., the radial coordinate) while dispersing it in energy.

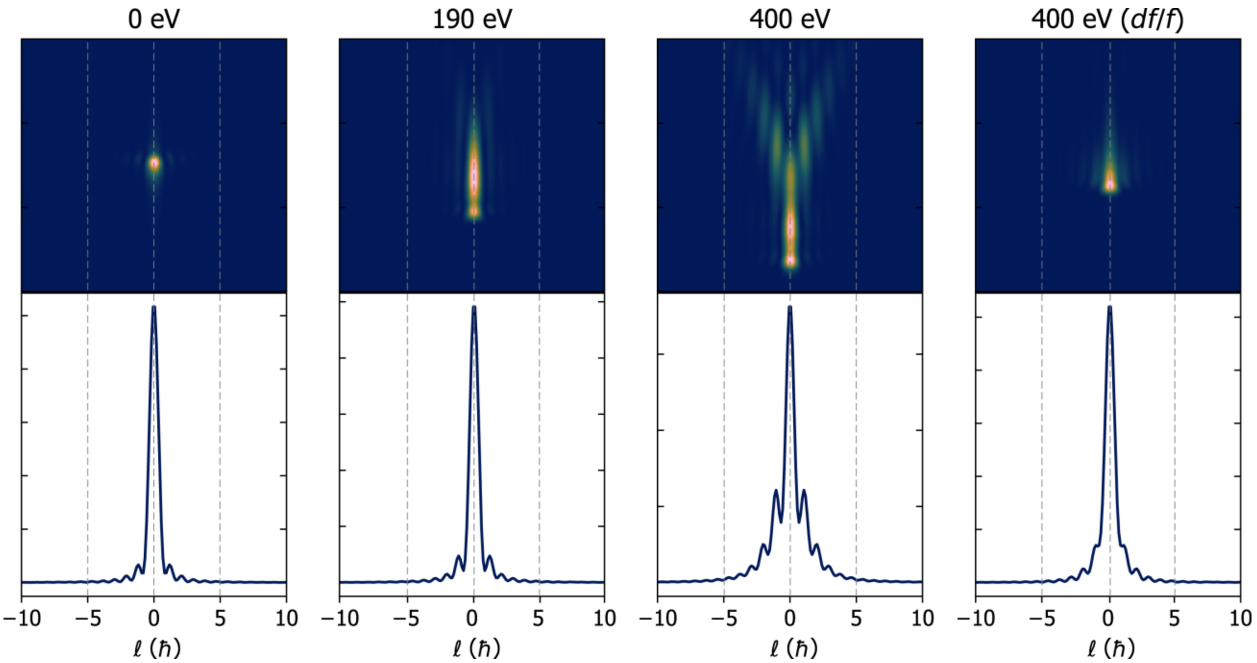

Supplementary Figure 5. Effects of chromatic aberration. (top row) Intensity maps of the Sorter psf as a function of the OAM quantum number ℓ (in units of ħ), shown for different energy losses: 0 eV, 190 eV, 400 eV, and 400 eV with defocus correction (df/f). (bottom row) Corresponding line profiles showing the OAM intensity distribution (dark blue lines). The broadening and distortions at higher energy-losses (e.g., 400 eV) highlight the sorter's chromatic aberration effects, which are partially mitigated by defocus correction.

The calculations were performed at different energies, with zero-loss serving as a reference. For the B K-edge (190 eV) used in this study, we observe that the effect of chromatic aberration is mainly along the radial direction and therefore has negligible influence on OAM resolution. This suggests that we have at least 400 eV energy range, centred on the energy at which the sorter is aligned, free from significant chromatic aberration. Given the typical energy dispersion in EELS experiments, this window is sufficiently large for most applications.

To observe a noticeable chromatic effect, an energy loss as high as 400 eV must be considered. Even at such large energy loss, it is worth noting that the impact of chromatic aberration is comparable to a simple defocus[5] (df/f). Consequently, it can be largely compensated for by a small defocus adjustment (e.g., $df/f = 0.2\%$ of the objective lens focal distance f at 400 eV).

In conclusion, chromatic aberration is not a significant factor, and we have neglected its effect in the present application. It becomes relevant only if the beam is not well centred on the objective lens, as this would introduce a chromatic shift that could potentially disrupt the alignment of the sorter. However, beam centring is an integral part of the standard microscope alignment (rotation centre alignment).

Supplementary Note 5. Quantitative evaluation of the quality of OAM decomposition.

The small residual peak at 190 eV in the $m = \pm 1$ (σ*) spectrum in Figure 4b of the main text can be due to several causes: i) a small tilting or damaging of the sample during acquisition, ii) a drifting of the alignment condition of the device during acquisition, iii) residual channelling effects or iv) a limitation of the deconvolution. If the source of discrepancy is a residual channelling effect, not considered by the simplified (kinematic) model of propagation used our model-based procedure, more rigorous modelling based on a full multislice could be explored in the future.

However, despite this peak, the overall intensities in the features agree well between the experiment and the theoretical predictions. To quantify the level of agreement, we report some parameters:

1) The ratio between the π* peak maximum and the σ* peak maximum is 1.73 for the experiment and 1.79 for the theoretical calculation in Figure 4b,c of the main text, with a discrepancy of 4%.

2) A metric for the similarity between two signals is given by the normalized cross-correlation (NCC). The NCC between two vectors $\mathbf{a}$ and $\mathbf{b}$ (the spectra to compare in our case) is defined as:

$$\mathrm{NCC}(\mathbf{a},\mathbf{b}) = \frac{\mathbf{a}\cdot\mathbf{b}}{ab} = \frac{\sum_i a_i b_i}{\sqrt{\sum_i a_i^2}\sqrt{\sum_i b_i^2}}, \tag{9}$$

and gives a quantitative measurement of the similarity between the two vectors. Ideal match is +1 when $\mathbf{a} = \mathbf{b}$, orthogonality is 0, and complete anticorrelation $\mathbf{a} = -\mathbf{b}$ is -1. The NCC between experimental and theoretical π* and σ* components (full lines in Figure 4b of the main text) in the interval 185 – 220 eV, is 0.93 for π* and 0.88 for σ*, respectively. We excluded higher energies due to deviations in the ab-initio DFT calculations for high levels empty states. These values indicate high correlation between the experimental and theoretical spectra and are remarkable for a proof-of-concept demonstration obtained with a custom-built experimental setup.

3) If we assume that the peak at $m = \pm 1$ at 190 eV is due to a cross-talk between the $m = 0$ and $m = \pm 1$ channels, the experimental intensity at $m = \pm 1$ can be written as $\mathrm{I_{exp}}(m = \pm 1) = c\mathrm{I_{th}}(m = 0)$, while $\mathrm{I_{exp}}(m = 0) = (1 - 2c)\,\mathrm{I_{th}}(m = 0)$, with $\mathrm{I_{th}}$ the intensity from calculations. The crosstalk coefficient in the deconvoluted spectrum results:

$$c = \frac{\mathrm{I_{exp}}(m=1)}{\mathrm{I_{exp}}(m=0) + 2\mathrm{I_{exp}}(m=1)}. \tag{10}$$

In the spectra of Figure 4b, $c = 11\%$. This incertitude is acceptable for many applications. For example, if we assume conservatively that the cross talk between +1 and -1 is $\leq 0.5c = 6\%$, which is sufficient to estimate magnetic dichroism values that may reach ≥18%[6].

Finally, if the source of discrepancy is a residual channelling effect, not considered by the simplified (kinematic) model of propagation used our model-based procedure, more rigorous modelling based on a full multislice could be explored in the future.

Supplementary Note 6. OAM-EELS experiment in ideal experimental conditions.

Technological limitations in our current experimental setup partly exacerbate the delocalization effect, but this issue can be mitigated through straightforward improvements in the fabrication of the first sorting element, which is simply a needle. Enhancing the precision of this component will enable the use of a larger beam-forming aperture, leading to a reduction in probe size while simultaneously increasing the beam current. This, in turn, will allow for shorter acquisition times, thereby minimizing sample drift and improving the overall stability and accuracy of the measurements. Moreover, the possibility of scanning the beam would enable localizing the probe on the atomic columns.

We can simulate this condition using the simplified delocalisation model, as reported in Supplementary Figure 6. Using a 7 mrad probe (which is the standard value for uncorrected microscope at 300 keV) and locating the beam probe directly on top of an atomic column is sufficient to drastically reduce the delocalisation effect and obtain a clean spectrum where only the $m = -1, 0, +1$ components are present. Further reducing the probe size using a 15 mrad aperture (which is instead a common value for C_s corrected microscopes) further improve the spectrum quality.

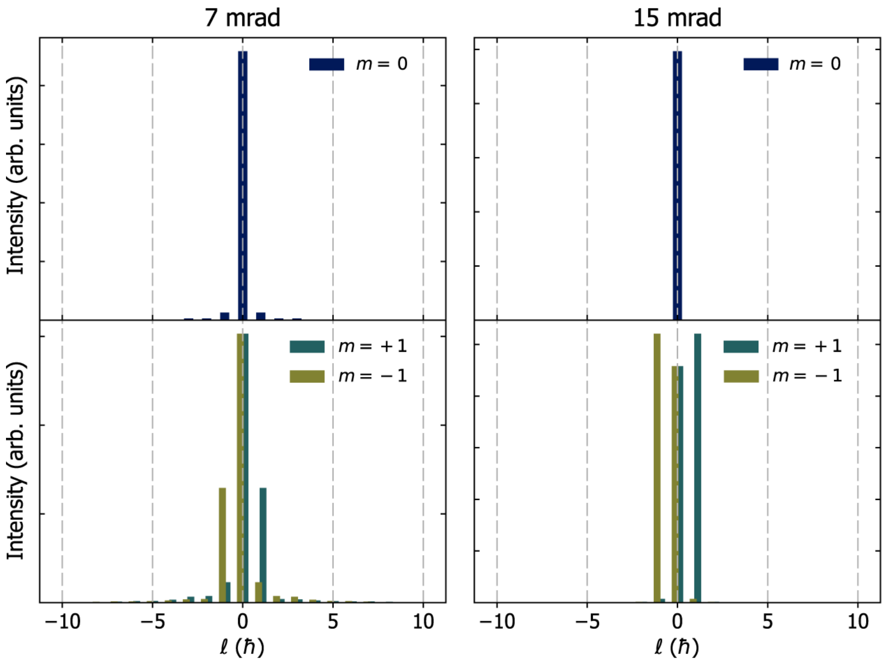

Supplementary Figure 6. Simulated OAM profiles in the case of negligible drift. OAM profiles $\Gamma_m(\ell)$ for $m = 0$ (dark blue bars) and $m = \pm 1$ ($m = +1$ teal bars, $m = -1$ olive green bars), calculated for a stationary sample with the probe positioned directly on top of an atomic column. The simulations are performed for convergence angles of $\alpha = 7$ mrad (left) and $\alpha = 15$ mrad (right).

In these conditions, the interpretation of OAM spectra is more direct, due to a narrower OAM spread, but a degree of mixing between components is unavoidable and model-based deconvolution remain essential.

Supplementary References

1. Hu, X., Sun, Y. & Yuan, J. Multivariate statistical analysis of electron energy-loss spectroscopy in anisotropic materials. *Ultramicroscopy* **108**, 465–471 (2008).
2. Schattschneider, P., Nelhiebel, M. & Jouffrey, B. Density matrix of inelastically scattered fast electrons. *Phys. Rev. B* **59**, 10959 (1999).
3. Schattschneider, P. *Linear and Chiral Dichroism in the Electron Microscope*. *Linear and Chiral Dichroism in the Electron Microscope* (Jenny Stanford Publishing, New York, 2012). doi:10.1201/b11624.
4. Verbeeck, J., Schattschneider, P. & Rosenauer, A. Image simulation of high resolution energy filtered TEM images. *Ultramicroscopy* **109**, 350–360 (2009).
5. Rotunno, E. *et al.* Alignment of electron optical beam shaping elements using a convolutional neural network. *Ultramicroscopy* **228**, 113338 (2021).
6. Rotunno, E. *et al.* Orbital angular momentum resolved electron magnetic chiral dichroism. *Phys. Rev. B* **100**, 224409 (2019).
